# Sex-Related Differences in Catheter Ablation for Patients With Atrial Fibrillation and Heart Failure

**DOI:** 10.3389/fcvm.2020.614031

**Published:** 2020-12-14

**Authors:** Tamanna Chibber, Adrian Baranchuk

**Affiliations:** Division of Cardiology–Kingston Health Sciences Centre, Queen's University, Kingston, ON, Canada

**Keywords:** atrial fibrillation, heart failure, catheter ablation, sex-related differences, gender differences

## Abstract

The coexistence of atrial fibrillation and heart failure significantly increases the risk of all-cause mortality and heart failure hospitalizations. Sex-related differences in all patients undergoing atrial fibrillation catheter ablation include the referral of fewer women for catheter ablation (15–25%), older age of women at ablation, and higher risk of post-ablation recurrence of atrial fibrillation. We searched the existing literature for sex-related differences in patients undergoing atrial fibrillation catheter ablation with a focus on heart failure. Randomized controlled trials assessing atrial fibrillation catheter ablation in patients with heart failure have demonstrated a significant reduction in all-cause mortality and heart failure hospitalizations. Within the eight existing randomized controlled trials on heart failure with reduced ejection fraction, women composed a small proportion of the study population. Only two studies (CASTLE-AF and AATAC-HF) specifically assessed the effect of gender on outcome and showed no difference in post-ablation outcomes. Registry data-based studies assessing sex-related differences in atrial fibrillation catheter ablation in heart failure reveal that women are half as likely as men to undergo ablation. Conflicting data exist on the interaction of gender and heart failure as they may affect peri-ablation and post-ablation long-term outcomes such as atrial fibrillation recurrence or heart failure hospitalizations. In conclusion, existing studies provide insight into the gender-based differences in patients undergoing catheter ablation for atrial fibrillation as it pertains to heart failure. Further prospective studies with higher proportions of female participants are required to accurately determine gender-based differences in this population.

## Introduction

In patients with clinically overt heart failure (HF), atrial fibrillation (AF) affects ~15–30% of patients (1). Concomitant occurrence of AF and HF significantly increases the risk of all-cause mortality, HF hospitalizations, and thromboembolism (1–3). Existing randomized controlled trials evaluating the effect of catheter ablation (CA) on outcomes in patients with AF and heart failure with reduced ejection fraction (HFrEF) have demonstrated a significant reduction in all-cause mortality and HF hospitalizations (4–11). The largest randomized controlled trial—CASTLE-AF (4)—demonstrated significant improvement in left ventricular ejection fraction (LVEF), all-cause mortality, and HF hospitalization with AF-CA in patients with LVEF ≤35% as compared to the oral rate or rhythm control (4). It is not clear if and how the results of the trials of AF-CA in HF are applicable to women in particular. In the general AF population, epidemiologic studies have demonstrated that women are more likely to have adverse events from anti-arrhythmic drugs, higher stroke risk, more disabling strokes, and higher cardiovascular mortality compared with men (2, 12–15). Yet, generally, women with AF are less likely to undergo CA (15–17). Proposed reasons for this include more procedural difficulty due to non-pulmonary vein triggers and atrial fibrosis, older age and presence of more underlying comorbidities. Women may have up to a 2.3–fold increased risk of procedural complications, including tamponade, vascular site complications and longer post-procedural hospitalization (17–20). The aim of this non-systematic review is to amalgamate the knowledge on gender differences in patients undergoing AF-CA with a focus on HF.

## Methods and Materials

A non-systematic review of the existing literature on sex-related differences in CA for AF in HF has been conducted. We searched PUBMED, EMBASE, and MEDLINE looking for the most relevant existing literature on this topic. MESH terms included: atrial fibrillation, catheter ablation, gender/sex differences, heart failure, and their combinations. Studies that were not in humans or in English were not considered for this review. Studies combining arrhythmias where atrial fibrillation data could not be separately assessed were also eliminated. The papers obtained by the search were reviewed by the two authors for their relevance to the topic. Disagreements were solved by consensus.

## Results

### AF-CA in *HFrEF*: Gender Effect

Eight randomized controlled trials assess the effect of AF-CA in patients with HFrEF. The female population in these studies ranges from 4 to 27%. Table 1 summarizes the eight randomized controlled trials, including the ratio of men to women in these trials (4–11). Two trials assess the gender effect on outcomes. The AATAC trial (8) comparing AF-CA with amiodarone in patients with LVEF ≤40% demonstrated significantly less recurrence of AF (recurrence free in CA 70 vs. 34% in amiodarone group; *p* < 0.001), reduced hospitalization (CA 31% vs. amiodarone 57%; *p* < 0.001), and reduced mortality (CA 8% vs. amiodarone 18%; *p* = 0.037). Gender did not affect AF recurrence, but women only composed 25% of the study population (8). In CASTLE-AF (4)—the largest randomized controlled trial in patients with AF and LVEF ≤35%—patients were randomized to CA or medical therapy (rate or rhythm control) with follow-up over 37.8 months. AF-CA demonstrated significantly greater maintenance of sinus rhythm (CA 63.1% vs. control 21.7%; *p* < 0.001), improvement in LVEF (CA 8% increase vs. control 0.2% increase; *p* = 0.005), and reduction in the composite outcome of all-cause mortality and HF hospitalization (CA 28.5% vs. control 44.6%; *p* = 0.006). Subgroup analysis to determine the effect of gender did not demonstrate a statistically significant difference in the primary outcome of death or hospitalization for HF (female HR 0.93 vs. men HR 0.58; *p* = 0.36). However, there is a trend toward men benefiting more from ablation while women appeared to have no significant benefit. The interpretation of this analysis is limited by the low proportion of women in both treatment arms (13% CA vs. 16% medical therapy) (4). The most recent trial in the AF and HFrEF population—AMICA (11)—did not demonstrate improvement in LVEF or symptoms with CA. Notably, women made up only 10% of the study population, and no gender-based differences in outcomes were assessed (11).

**Table 1:** Summary of randomized controlled trials on atrial fibrillation catheter ablation in patients with heart failure with reduced ejection fraction.

**Trial (year of publication)**	***N***	**Gender M:F ratio**	**Inclusion criteria**	**Treatment arm**	**Primary end point**	**FU (months)**	**Prominent findings**
PABA-CHF (2008) (5)	81	74:7	Paroxysmal or persistent AF, NYHA II–III, and LVEF ≤40%	PVI (±additional ablation) vs. CRT plus AV node ablation	Composite of LVEF (echo), 6MWD or MLWHF score	6	88% AF-free survival in ablation arm (71% off AAD); significant increase in LVEF (+8 vs. −1%), functional capacity, QOL
MacDonald et al. (2011) (6)	41	32:9	Persistent AF, NYHA II–IV, and LVEF <35%	PVI (±additional ablation) vs. pharmacological rate control	LVEF change (MRI)	6	50% AF-free survival in ablation arm (50% off AAD); non-significant increase in LVEF (significant if SR: +10 vs. +1%), functional capacity, QOL
ARC-HF (2013) (10)	52	45:7	Persistent AF, NYHA II–IV, and LVEF ≤35%	PVI (±additional ablation) vs. pharmacologic rate control	Change in peak oxygen consumption	12	88% AF-free survival in ablation arm (84% off AAD); significant improvement in peak VO2, QOL, BNP; non-significant increase in LVEF (+11 vs. +5%), 6MWD
CAMTAF (2014) (7)	50	48:2	Persistent AF, NYHA II–IV, and LVEF <50%	PVI (±additional ablation) vs. pharmacologic rate control	LVEF change (echo)	6	81% AF-free survival in ablation arm (81% off AAD); significant improvement in LVEF (+8 vs. −3%), functional capacity, QOL, BNP
AATAC-AF (2016) (8)	203	151:52	Persistent AF, NYHA II–III, LVEF ≤40%, and DC-ICD/CRT-D	PVI (±additional ablation) vs. amiodarone	AF-free survival	24	70% AF-free survival in ablation arm vs. 34% in amiodarone arm; significant improvement in LVEF (+8 vs. +6%), mortality (8 vs. 18%), hospitalization (31 vs. 57%), QOL
CAMERA-MRI (2017) (9)	68	60:6	Persistent AF, NYHA II–IV, LVEF ≤45%, and idiopathic cardiomyopathy	PVI + posterior box isolation vs. pharmacologic rate control	LVEF change (MRI)	6	75% AF-free survival in ablation arm (56% off AAD); significant improvement in LVEF (+18 vs. +14%), LVEF normalization ≥50% (58 vs. 9%); LGE- predicted LVEF improvement, normalization
CASTLE-AF (2018) (4)	363	311:52	Paroxysmal or persistent AF, NYHA II–IV, LVEF ≤35%, and DC-ICD/ CRT-D with remote monitoring	PVI (±additional ablation) vs. pharmacologic rate (70%) or rhythm control (30%)	Composite of HF hospital-ization or all-cause mortality	60	63 vs. 22% in SR at 5 years; significant improvement in LVEF (+8 vs. 0%), all-cause mortality or HF hospitalization (28 vs. 44%), all-cause mortality (13 vs. 25%), cardiovascular mortality (11 vs. 22%), HF hospitalization (21 vs. 36%)
AMICA (2019) (11)	140	126:14	Persistent AF, LVEF ≤35%, ICD/CRT-D	PVCI vs. optimal medical therapy (rate, rhythm or AV nodal ablation)	LVEF increase	12	73.5 vs. 50% in SR at 1 year; no significant increase in LVEF (8.8 vs. 7.3%), NT-proBNP, 6MWT, QOL

### AF-CA in *HFpEF*: Gender Effect

In patients with HF with preserved ejection fraction (HFpEF), only retrospective studies have assessed the effect of AF-CA. The most recent retrospective analysis of 85 patients with HFpEF (EF > 50%) and previous hospitalization with AF and HF, showed that AF-CA reduced HF hospitalization compared to pharmacotherapy (rate or rhythm control) over 2 years of follow-up. This cohort included only 35% women and gender based effects on outcomes were not assessed (21). In another 2018 retrospective study of 230 patients with AF and HF who underwent AF-CA, patients were subdivided into HFpEF (58.8%) and HFrEF (42.2%). CA showed similar effectiveness in both groups. Interestingly, women were 31.3% of the study population and were significantly more likely to have HFpEF (42.1%) as opposed to HFrEF (16.5%) but outcomes were not analyzed for gender effect (22).

### AF-CA in HF: Gender Effect in Registry Data

Given the limited gender-based data available in trials focusing on AF-CA and HF, studies based on registry datasets provide more insight into gender-related differences. In a Quebec cohort of 101,931 patients with AF and HF only 432 had undergone AF-CA. While 51.4% of the AF and HF cohort was female, only 25.6% of the CA population was female. In the general AF-HF cohort, women were older and had less frequent comorbidities, ICDs, CRTs, and use of medications, while men were younger and had less hypertension, valvular disease, and prior stroke. In the cohort of patients that underwent CA, there were no significant gender differences in age or comorbidities. Adjusting for advanced age and multiple comorbidities, women were approximately half as likely to undergo CA (23). In a 2018 retrospective cohort analysis of 54,645 patients with AF or atrial flutter and HF, 6,443 patients underwent left atrial CA. Of this cohort, 37.5% were female, who were significantly older than men (women 69 years old vs. men 62.7 years old; *p* < 0.001) and had significantly more comorbidities (*p* < 0.001). Women had significantly longer length of hospital stay (women 6 days vs. men 4.6 days; *p* < 0.001), vascular access complications (2.7 vs. 0.7%; *p* < 0. 001) and cardiac tamponade (1.5 vs. 0.5%; *p* < 0.001) (24). In another cohort of 10,966 patients who underwent AF-CA, compared with those patients without HF, patients with HF were more likely to be women (41 vs. 37.3%; *p* = 0.002). While the study demonstrated a significant reduction in all-cause hospitalization up to 4 months post CA in the HF and non-HF groups, the effect was more pronounced in the HF group. Outcomes were not stratified according to gender (25).

### AF-CA General Population

#### Greater Female Baseline Prevalence of HF

Broadening assessment to registry data in the general AF-CA population, recent studies provide further insight. In a cohort of 1,060 patients with AF-CA under the age of 60, 21% were females. Women were significantly older than men (women 50.8 years old vs. men 49.5 years old) and were more likely to have HF (*p* = 0.017), specifically, diastolic dysfunction (*p* < 0.01). Women showed significantly greater AF recurrence (39% for women vs. 27% for men; *p* < 0.001), but the interaction of gender and HF was not assessed (26). Using the FIRE and ICE study database, 750 patients with symptomatic paroxysmal AF refractory to anti-arrhythmic drugs underwent CA. The cohort included 39% women, who were older (age 64 years old for women vs. 57 years old for men), and had more HF at baseline. Women had significantly more AF recurrence, specifically a 37% increased risk of arrhythmia recurrence. However, a history of HF did not further affect this gender-based difference (27). In another cohort of 54,597 patients with AF-CA, 37.7% were female. Women were older, had significantly more comorbidities, specifically a greater prevalence of HF than men (women 17% vs. men 15.7%; *p* < 0.0001). Importantly, it identified a significantly higher 30-day post-ablation readmission rate for women than men (13.4 vs. 9.4%; *p* < 0.0001), with HF being the second leading cause of readmission accounting for 13% of all readmissions. However, a history of HF did not further influence the gender based difference in all-cause readmission (28).

#### No Baseline Gender Difference in HF Prevalence

In a Chinese cohort of 1,410 patients who underwent AF-CA, 31.9% were women who were older and had more paroxysmal AF. There was no significant gender difference in the baseline prevalence of HF (women 5% vs. men 5.3%; *p* = 0.75). While the study did not show any gender-related differences with respect to in-hospital complications or early or late recurrence of AF, women with AF recurrence were more likely to have had a previous history of HF (recurrence CHF 10.1% vs. no recurrence CHF 3.6%; *p* < 0.01) (29). In a prospective, multicenter, observational study of 5,010 consecutive patients undergoing AF-CA, women constituted 27.3% of the study population, were significantly older, and had a lower prevalence of non-paroxysmal AF. At baseline, there was no difference between men and women in HF prevalence (women 14% vs. men 12.9%). Women experienced significantly higher 3-year AF recurrence. Peri-procedurally, there was no significant gender-based difference in HF decompensation (women 0.37% vs. men 0.33%; *p* = 0.85). However, the 3-year incidence of HF hospitalizations tended to be higher in women (2.2% for women vs. 1.5% for men; *p* = 0.066). After adjusting for confounders, being female was an independent predictor for HF hospitalization (adjusted HR 2.17; *p* = 0.0014) (30).

In a meta-analysis of randomized controlled trials and large prospective observational studies to compare sex-related differences in patients undergoing cryoballoon vs. radiofrequency ablation, no effect of HF or LV systolic dysfunction (LVEF < 45%) was identified in either gender on peri-procedural complications, procedural/fluoroscopy time, or the combined outcome of arrhythmia recurrence, reablation, or reinitiation of medications up to 3 years of follow-up (31). Furthermore, 674 patients undergoing AF-CA from the AXAFA-AFNET 5 study, made consisted of 33% women, who were significantly older and more often had paroxysmal AF but were not otherwise more comorbid than men. At baseline, there were no gender-based differences in HF prevalence, but there was a trend toward women having more symptomatic NYHA II-III CHF (28.2% for women vs. 21.5% for men; *p* = 0.07). While there was no sex-related difference in maintenance of sinus rhythm, the effect of HF or HF as an outcome was not reported (32). Another systematic review and meta-analysis of observational studies included 151,370 patients undergoing AF-CA, of which 34% were women. Baseline characteristics and results were divided into two outcomes: freedom from AF/atrial tachycardia (AT) recurrence and complications (stroke/TIA, all-cause mortality). For the demographic of freedom from AF/AT recurrence, there were no baseline differences in the prevalence of HF and women were found to have a lower rate of freedom from AF/AT recurrence. In the demographic of complications, women had significantly less HF at baseline (23.8% for women vs. 25.5% for men; *p* = 0.0014) and demonstrated a trend toward an increased risk of stroke/TIA and all-cause mortality compared with men. Women were also more likely to experience pericardial effusion/tamponade, major bleeding, and pacemaker implantation. The exact interaction of gender and HF on these outcomes was not evaluated, although LVEF was not found to have an effect on freedom from AF/AT or stroke/TIA incidence in either gender (33).

## Discussion

In our review, we report that women are significantly underrepresented in trials assessing the effect of AF-CA in HF. Women with AF and HF undergoing CA are older with different comorbidities than men such as stroke or valvular heart disease. Within the limited available information, discrepancy exists on the interaction of gender and HF for AF-CA with respect to peri- and post-ablation outcomes.

Women are more likely to have AF and HF but are half as likely to undergo CA despite adjusting for age and comorbidities. Moreover, women are underrepresented compared to men in both randomized controlled trials and registry based cohort studies of patients with AF and HF (23, 25). This finding is also evident in many general AF-CA registry-based studies where there is no gender-based difference in the prevalence of HF, suggesting that despite the fact that women have more AF and HF, they are not equally being referred for CA (29–33). This gender discrepancy has been demonstrated in the general AF population undergoing CA where <30% of the CA population is female (15–17). Only two of the existing eight randomized trials of AF-CA in HFrEF assess for the effect of gender on outcomes. While gender did not have an effect on outcomes in either trial, the validity of the analysis is limited by the poor representation of women in both trials (4, 8). The limited number of women in these HFrEF trials may be explained by the finding from existing literature that men have a higher incidence of HFrEF and women with AF are more likely to have HFpEF (22, 34). However, even the few small trials of AF-CA in HFpEF include significantly fewer women than men and do not stratify outcomes for gender effect (21, 22).

While women with AF and HF are generally older than men, among those patients who undergo AF-CA there may not be an age difference between men and women. This suggests that apart from gender alone, older age may be another deterring factor in referring women with AF and HF for CA. This can possibly be mitigated by earlier referral of women for AF-CA, especially as previous studies have demonstrated that women are referred later for CA (35). Interestingly women with AF and HF are more likely to have valvular disease and prior stroke yet these differences are often not reflected in the population undergoing CA (23). Valvular heart disease particularly may be a factor that limits the efficacy of catheter ablation, which may again prevent women from being referred for CA (36). When women undergoing AF-CA in HF are older and more comorbid than men, women have a significantly greater length of post-procedural hospital stay, vascular access complications, and cardiac tamponade (24). Some discrepancy does exist with respect to peri-procedural complications, with some data suggesting no effect of HF or LV dysfunction on peri-procedural complications for either gender, nor any gender difference in peri-procedural HF occurrence (30, 31). In the general AF population undergoing CA, some studies have found women to have higher peri-procedural complications (17–20, 33). Anatomical differences, such as smaller heart size in women, may be factors that affect catheter manipulation in the heart chambers (35). Such an emerging finding may be another factor contributing to women being referred less often for AF-CA.

In the general AF-CA cohorts, there is a significant discrepancy in the effect of gender and HF on the efficacy of AF-CA. In some cohorts where women are older and more likely to have HF at baseline, women have significantly more AF recurrence post CA. However, the independent effect of a history of HF on this gender difference could not be consistently established, as some chorts even demonstrated no gender-based difference in AF recurrence in the general AF-CA cohort (26–29, 31, 33). Conflicting data also exist with respect to post-ablation readmission outcomes. In one cohort where women have a higher HF prevalence, women demonstrate a greater rehospitalization rate for up to 30 days post CA, with HF accounting for 13% of all readmissions (28). Meanwhile, another cohort study where women were more likely to have HF at baseline demonstrated lower post-ablation all-cause hospitalizations up to 4 months post CA (25). Furthermore, a cohort study with no gender-based difference in baseline prevalence of HF demonstrated significantly higher HF hospitalizations for up to 3 years post-CA in women (30). From these studies it is difficult to ascertain the direct interaction of gender and HF on the efficacy and outcomes of AF-CA.

## Conclusion

We report that in patients with AF and HF, women are significantly underrepresented in randomized controlled trials and cohort studies assessing the effects of AF-CA. Independent of other factors, female sex and older age were both factors that limited the inclusion of women with HF in studies assessing the efficacy of AF-CA. Conflicting evidence exists on the interaction of HF and gender with respect to outcomes at the time of and after AF-CA. Going forward, trials on AF-CA in HF should work toward including more female participants and at least assessing for the effect of gender on outcomes as there may be significant gender-based differences. Future research should also attempt to explicitly determine the factors that lead to the disparities between men and women from referral for AF-CA in HF to degree of benefit or harm from the ablation.

## Author Contributions

All authors listed have made a substantial, direct and intellectual contribution to the work, and approved it for publication.

## Conflict of Interest

The authors declare that the research was conducted in the absence of any commercial or financial relationships that could be construed as a potential conflict of interest.
